# Individuals with chronic low back pain have greater difficulty in engaging in positive lifestyle behaviours than those without back pain: An assessment of health literacy

**DOI:** 10.1186/1471-2474-12-161

**Published:** 2011-07-15

**Authors:** Andrew M Briggs, Joanne E Jordan, Peter B O'Sullivan, Rachelle Buchbinder, Angus F Burnett, Richard H Osborne, Leon M Straker

**Affiliations:** 1 School of Physiotherapy and Curtin Health Innovation Research Institute, Curtin University, Australia; 2 Monash Department of Clinical Epidemiology at Cabrini Hospital, School of Public Health and Preventive Medicine, Monash University, Australia; 3 Department of Sports Science and Physical Education, Chinese University of Hong Kong, Hong Kong; 4 School of Exercise, Biomedical and Health Sciences, Edith Cowan University, Australia; 5 Public Health Innovation, Deakin Population Health Strategic Research Centre, School of Health and Social Development, Deakin University, Australia

**Keywords:** health literacy, low back pain, health information, HeLMS, self-management

## Abstract

**Background:**

Despite the large volume of research dedicated to understanding chronic low back pain (CLBP), patient outcomes remain modest while healthcare costs continue to rise, creating a major public health burden. Health literacy - the ability to *seek, understand *and *utilise *health information - has been identified as an important factor in the course of other chronic conditions and may be important in the aetiology of CLBP. Many of the currently available health literacy measurement tools are limited since they measure narrow aspects of health literacy. The Health Literacy Measurement Scale (HeLMS) was developed recently to measure broader elements of health literacy. The aim of this study was to measure broad elements of health literacy among individuals with CLBP and without LBP using the HeLMS.

**Methods:**

Thirty-six community-dwelling adults with CLBP and 44 with no history of LBP responded to the HeLMS. Individuals were recruited as part of a larger community-based spinal health study in Western Australia. Scores for the eight domains of the HeLMS as well as individual item responses were compared between the groups.

**Results:**

HeLMS scores were similar between individuals with and without CLBP for seven of the eight health literacy domains (p > 0.05). However, compared to individuals with no history of LBP, those with CLBP had a significantly lower score in the domain 'Patient attitudes towards their health' (mean difference [95% CI]: 0.46 [0.11-0.82]) and significantly lower scores for each of the individual items within this domain (p < 0.05). Moderate effect sizes ranged from *d *= 0.47-0.65.

**Conclusions:**

Although no differences were identified in HeLMS scores between the groups for seven of the health literacy domains, adults with CLBP reported greater difficulty in engaging in general positive health behaviours. This aspect of health literacy suggests that self-management support initiatives may benefit individuals with CLBP.

## Background

Low back pain (LBP) represents a major public health issue globally [1]. Despite the vast majority of individuals experiencing LBP at some point in their lives, only a modest proportion experience chronic LBP (CLBP) with ongoing disability [2,3]. Nonetheless, this subgroup consumes the majority of health resources related to spinal pain [4,5], highlighting an urgency to identify modifiable risk factors for chronicity and disability related to LBP, and to optimise the delivery of treatment approaches and health information for consumers and health providers [5]. Although a large volume of research has been undertaken to test the efficacy of treatment approaches for CLBP, only modest treatment effects have been observed and health expenditure continues to soar [6,7]. This trend may suggest that potentially important mediating factors are being overlooked. *Health literacy *may be one such factor.

Clinical guidelines for the management of CLBP stress the importance of adopting a biopsychosocial approach to management, where self-management through optimisation of pain and lifestyle behaviours are important for recovery [8,9]. Such an approach demands an active role from patients in management. However, adequate engagement from patients throughout this cycle of care assumes that an individual has sufficient health literacy [10,11] - that is, the capacity to *seek, understand *and *utlilise *health information [12]. Suboptimal health literacy in patients with other chronic health conditions such as asthma [13,14], diabetes [15] and rheumatoid arthritis [16-18] is associated with poorer condition knowledge [15,19] and limited self-management skills [13,20]. Health literacy, therefore, has important implications for health programmes and health service delivery models, particularly in the context of management of chronic health conditions.

In a recent community cohort study we observed that individuals with CLBP and those with no history of CLBP had functional health literacy scores which were similar and categorised as 'adequate' [21], when measured with the short-form Test of Functional Health Literacy in Adults (s-TOFHLA) - an instrument which measures only numeracy and reading comprehension within a healthcare context [22]. However, in a qualitative arm of that study involving participants with CLBP we identified that these individuals encountered several barriers in seeking, understanding and utilising LBP information [21]. For example, participants reported difficulty in finding reliable information about LBP management options within their local communities, understanding medical terminology, and implementing advice from health professionals when it was discordant with their beliefs or precluded by socioeconomic circumstances. Importantly, these barriers to optimal health literacy were not reflected in the s-TOFHLA scores, highlighting that the instrument was unable to capture these broader and clinically relevant elements of health literacy [21]. This limitation is also reflected in other health literacy measurement tools [23]. Whether individuals with no history of CLBP also experience barriers in seeking, understanding and utilising general health information is uncertain.

Recently, the Health Literacy Measurement Scale (HeLMS) was developed to measure elements of health literacy beyond numeracy and reading comprehension and thereby overcome many of the perceived limitations of existing instruments [24]. The HeLMS instrument offers the advantage of being able to collect information about the broader elements of health literacy (see Table 1), which has only been undertaken in the past using time consuming qualitative methods [21,25,26]. In our earlier study, we examined some of these domains, specifically seeking, understanding and utilising health information [21]. The aim of the study described in this paper was to undertake a secondary investigation with the community cohort study we mentioned previously to further examine broad elements of health literacy among individuals with CLBP and those without a history of LBP using the HeLMS. We hypothesised that HeLMS scores would reflect poorer health literacy in individuals with CLBP, relative to individuals with no LBP, in areas that were comparable to those identified from our qualitative study and potentially identify additional aspects of poorer health literacy that were not identified in our qualitative study.

**Table 1:** Description of the domains of the HeLMS

Domain	Domain title	Domain description
**1**	Patient attitudes towards their health	This domain assesses an individual's ability to attend to their health needs as well as a willingness to change their lifestyle or adapt their behaviour to maintain their health.
**2**	Understanding health information	This domain focuses on an individual's ability to access and understand different formats of health information.
**3**	Social support	This domain assesses an individual's ability to seek social support so that they can manage their health. Social support refers to family, friends and broader community networks.
**4**	Socioeconomic factors: accessing healthcare services	This domain covers broader socioeconomic circumstances of an individual (financial resources) to be able to access health information and services.
**5**	Accessing General Practitioner (GP) healthcare services	This domain is concerned with an individual's ability to access GP healthcare services and knowing where to seek health information.
**6**	Communication with health professionals	This domain assesses an individual's ability to communicate with health professionals to get the information they want about their health.
**7**	Being proactive	This domain focuses on an individual's ability to be proactive in seeking and understanding information about their health.
**8**	Using health information	This domain refers to an individual's ability to understand and use information to make informed health decision and maintain their health.

## Methods

### Design

Participants in this study represent a subset of the Joondalup Spinal Health Study (JSHS) cohort. The JSHS has been described in detail elsewhere [21]. Briefly, the JSHS was established as a community-based cohort study in Perth, Western Australia, from November 2008 to examine familial associations in LBP. Potential participants were initially contacted through random dialling of residential phone numbers within a 10 km radius of the study centre using the Perth electronic telephone directory. Families were recruited on the basis of at least one adult and one child in the same family reporting significant LBP (pain families), and families where no member reported LBP in the last year (control families). The total sample size of the JSHS was N = 231 (adults = 151, children = 80). In this sub-study, adults of the original JSHS who previously reported either CLBP, that is pain persisting for 3 months or more (N = 56), or no history of LBP within the last 12 months (termed here as 'controls') (N = 61) were contacted again in August 2009 (4-9 months later) and invited to participate in a secondary study to further examine health literacy using the HeLMS. Of the 117 adults in the original JSHS cohort, 80 (68.4%) accepted the invitation to complete the HeLMS. Approval to conduct this secondary study was granted by the Curtin University Human Research Ethics Committee.

### Data collected

Data collected from questionnaires in the original JSHS are also reported in this paper for the purposes of describing the cohort which responded to the HeLMS and comparing them to the non-responders. Descriptions and psychometric properties of the tools used to collect data in the original JSHS have been described in detail previously [21]. In summary, data included:

• Demographics: age, gender.

• Clinical characteristics of LBP: The Nordic Musculoskeletal Pain Questionnaire [27] was used to define the boundaries of LBP using a body chart and prevalence estimates of LBP to determine group eligibility.

• Pain severity and impact: LBP intensity in the past week was quantified with a numeric pain rating scale (NRS) (0-10 where 0 = no pain and 10 = extreme pain) [28]. The impact of LBP was measured by asking the participants to indicate the number of LBP episodes in the last year (1-3, 4-10, >10 episodes), number of work days missed (0, 1-2, 3-7, 15-30, 181-365 days), their need to seek health professional advice (yes/no) and use medication (yes/no), and any interference with normal daily activities (yes/no) and recreational activities (yes/no).

• Disability related to LBP was measured using the Oswestry Disability Index (ODI) [29] and expressed as a percentage, where higher scores represent greater disability.

• Fear Avoidance was measured using the Fear Avoidance Beliefs Questionnaire (FABQ) [30]. Subscales for work (scored 0-42) and physical activity (scored 0-24) were derived, with higher scores indicating greater fear avoidance attitudes.

• Beliefs about back pain were measured using the Back Pain Beliefs Questionnaire (BBQ) (scored 9-45) [31]. Lower scores represent more negative beliefs about back pain.

• Pain catastrophizing was measured using the catastrophizing subscale of the Coping Skills Questionnaire (CSQ) (scored 0-24) [32]. Higher scores represent greater catastrophizing behaviours related to pain.

• Functional health Literacy was measured using the (s-TOFHLA) [22]. This tool measures two domains - reading comprehension and numeracy, based on respondents interpreting excerpts of text commonly encountered in hospital settings which were adapted to the Australian setting [33]. Higher scores (range 0-100) represent higher health literacy, with scores of ≥67 indicating adequate health literacy.

Participants with CLBP responded to all questionnaires while those with no history of LBP responded only to questions relating to demographics, the BBQ, CSQ and s-TOFHLA.

In this secondary study all participants were invited to respond to the HeLMS and report their highest level of education using a 7-point nominal scale, considering the reported association between health literacy and education level [34,35]. The HeLMS is based upon a health literacy conceptual framework from the patient perspective. This framework was developed through broad consultation with individuals with different healthcare experiences and consists of components that patients regard as important in seeking, understanding and utilising health information [26]. The HeLMS was developed using both exploratory and confirmatory factor analysis and was tested with a wide range of patients with construction (N = 333) and confirmatory (N = 350) samples drawn from both healthcare (primary and acute) and general community settings. The final measurement model, consisting of eight specific and independent constructs (domains), demonstrated good measurement properties (root means square error of approximation = 0.07 and standardised root mean squared residual = 0.05) [24]. Test-retest reliability and internal consistency across the domains has been established previously (ICC range: 0.73-0.96 and Cronbach's α range: 0.82-0.89) [24]. The domains include: patient attitudes towards health, understanding health information, social support, socioeconomic considerations, accessing general practitioner (GP) healthcare services, communicating with health professionals, being proactive, and using health information. Table 1 summarises the content of the domains.

The HeLMS consists of 29 items, each rated on a 5-point Likert scale. Questions relate to an individual's ability to *seek, understand *or *utilise *health information scored from 1 (unable to do) to 5 (able to do without any difficulty). To calculate scores in each domain, item scores (range: 1-5) were averaged.

### Data Analysis

Questionnaire data collected in the original JSHS were also compared between the groups in order to describe the cohort who participated in the secondary HeLMS study and to assess any responder bias. HeLMS domain scores were compared between individuals with CLBP and those with no history of LBP. Since each HeLMS domain represents an independent construct, eight comparisons were undertaken. Where a significant difference was identified for a given domain, the individual item responses were also compared between the groups. Responses for each item were also dichotomised as 'no difficulty' (ie a score of 5 on the Likert scale) or 'any difficulty' (ie a score of 1-4 on the Likert scale). Continuous data were analysed with independent t-tests and categorical data with chi square tests. For continuous data both parametric (t-tests) and non-parametric tests (Mann-Whitney tests) were performed given that some data were skewed. The conclusions were the same for both types of tests, therefore only the results of parametric tests are presented. Effect sizes between the groups for the HeLMS responses were expressed using Cohen's *d*. Results were considered statistically significant where p ≤ 0.05. All data were analysed using SPSS Statistics 17.0 (SPSS Inc., Chicago, IL).

## Results

There were no differences in the distributions of age, gender and level of education between responders (n = 36 with CLBP and n = 44 controls) and non-responders (n = 20 with CLBP and n = 17 for controls) to the secondary survey in each group. Further, no differences were observed in clinical characteristics between responders and non-responders, other than the BBQ score for control participants where the non-responding controls had a significantly higher (more positive beliefs) BBQ score than responding controls (mean difference [95% CI]: -4.16 [-7.03 - -1.29], p = 0.006) (Table 2).

**Table 2:** Demographic and clinical characteristics of participants according to pain group (no LBP and CLBP) and according to participation in the secondary study (responder and non-responder)

Descriptor	No LBP		CLBP	
	Responder	Non-responder	Responder	Non-responder
N (% within pain groups)	44 (72.1)	17 (27.9)	36 (64.3)	20 (35.7)
Age (years), mean (SD)	37.8 (15.2)	40.5 (7.9)	42.2 (12.3)	37.0 (16.6)
Female, N (%)	25 (56.8)	12 (70.6)	24 (66.7)	10 (50.0)
Highest level of education, N (%)				
Never attended school	0 (0)		0 (0)	
Some primary school	0 (0)		0 (0)	
Completed primary school	0 (0)		0 (0)	
Some secondary school	0 (0)		2 (5.6)	
Completed secondary school	8 (18.2)		7 (19.4)	
Trade certificate or diploma	17 (38.6)		16 (44.4)	
University degree	19 (43.2)		11 (30.6)	
Age of onset of LBP (years), mean (SD)			26.9 (11.8)	21.4 (11.4)
Intensity of pain in last week, median (IQR) for NRS 0-10)			4 (3)	4 (4)
Episodes of LBP in the last year, N (%)				
1-3 episodes			2 (5.6)	0 (0)
4-10 episodes			10 (27.8)	7 (35.0)
> 10 episodes			24 (66.7)	13 (65.0)
Number of work days missed due to LBP, N (%)				
0 days			25 (69.4)	12 (63.2)
1-2 days			2 (5.6)	4 (21.1)
3-7 days			7 (19.4)	3 (15.8)
15-30 days			1 (2.8)	0 (0)
181-365 days			1 (2.8)	0 (0)
no response			0 (0)	1 (0)
Impact of LBP, N (%) responding 'yes'				
Seeking health professional advice			16 (44.4)	10 (50.0)
Using medication for pain			18 (50.0)	5 (25.0)
LBP interfering with normal activities			22 (62.9)	11 (55.0)
LBP interfering with recreational activities			23 (63.9)	9 (45.0)
ODI score (%), mean (SD)			18.3 (10.2)	15.3 (8.8)
FABQ-physical activity score [0-24], mean (SD)			15.1 (4.9)	13.7 (5.6)
FABQ-work score [0-42], mean (SD)			10.6 (9.6)	9.9 (7.8)
BBQ score [9-45], mean (SD)	26.3 (5.9)	30.4 (4.4)*	26.9 (5.6)	28.3 (6.6)
CSQ catastrophizing score [0-24], mean (SD)	5.1 (4.6)	4.4 (4.9)	6.1 (5.4)	6.3 (4.2)
S-TOFHLA numeracy score [0-28], mean (SD)^^^	27.8 (1.1)	27.6 (1.8)	27.0 (3.0)	27.7 (1.6)
S-TOFHLA reading comprehension score [0-72], mean (SD)^^^	70.4 (1.9)	70.3 (2.3)	70.8 (1.8)	71.0 (1.2)
S-TOFHLA total score [0-72], mean (SD)^^^	98.4 (1.9)	97.8 (2.6)	97.6 (4.1)	98.7 (2.2)

NRS = numeric rating scale

* significant difference between responder and non-responder (p < 0.01)

^ The s-TOFHLA score is usually reported as a categorical outcome (inadequate (0-53), marginal (54-66), adequate (67-100). Here we report the data as continuous for finer level comparison.

Participants with CLBP had a significantly lower score in the HeLMS domain 1: 'Patient attitudes towards their health' compared to those with no history of LBP (mean difference [95% CI]: 0.46 [0.11-0.82], p = 0.01), and a moderate effect size was observed (*d=*0.65). No other differences were observed between groups for the seven other HeLMS domains. Compared to individuals with no history of LBP, significantly lower scores were identified for participants with CLBP for all individual items within domain 1 ('Patient attitudes towards their health') (p < 0.05) (Table 3) and moderate effect sizes were observed across the items (*d = *0.47-0.57). The proportion of people who experienced any level of difficulty was significantly higher in the CLBP group compared to the no LBP group for all items in domain 1: item 6 (72.2% vs. 47.7%, p = 0.03), item 14 (47.2% vs. 25.0%, p = 0.04), item 25 (47.2% vs. 22.7%, p = 0.02), and item 27 (58.3% vs. 36.4%, p = 0.05).

**Table 3:** Mean score, standard deviation (SD) and range for each domain and item of the HeLMS for the control (No LBP) and chronic low back pain (LBP) groups

Domain	Descriptor	*No LBP * Mean (SD), [min-max]	*Chronic LBP * Mean (SD), [min-max]	Mean difference (95% CI)	Cohen's (95% CI)
1	Patient attitudes towards their health	4.52 (0.71), [2.50-5.00]	4.01 (0.89), [2.00-5.00]	0.46 (0.11 - 0.82)*	0.65 (0.44 - 0.94)
2	Understanding health information	4.86 (0.36), [3.25-5.00]	4.80 (0.43), [2.75-5.00]	0.06 (-0.12 - 0.24)	0.15 (0.05 - 0.30)
3	Social support	4.72 (0.50), [2.75-5.00]	4.54 (0.75), [1.50-5.00]	0.17 (-0.11 - 0.46)	0.29 (0.14 - 0.54)
4	Socioeconomic considerations	4.70 (0.56), [2.33-5.00]	4.60 (0.54), [3.00-5.00]	0.10 (-0.15 - 0.34)	0.18 (0.02 - 0.36)
5	Accessing general practitioner healthcare services	4.90 (0.25), [3.75-5.00]	4.90 (0.34), [3.50-5.00]	0.02 (-0.12 - 0.15)	0.00 (-0.07 - 0.11)
6	Communicating with health professionals	4.68 (0.52), [3.00-5.00]	4.53 (0.59), [3.00-5.00]	0.15 (-0.09 - 0.40)	0.27 (0.12 - 0.47)
7	Being proactive	4.64 (0.54), [3.00-5.00]	4.50 (0.66), [2.67-5.00]	0.15 (-0.11 - 0.42)	0.24 (0.08 - 0.45)
8	Using health information	4.84 (0.34), [3.75-5.00]	4.71 (0.49), [2.75-5.00]	0.13 (-0.06 - 0.31)	0.32 (0.22 - 0.48)
					
**Item [domain]**	**Question: "Are you able to....."**				
6 [1]	Make time for things that are good for your health?	4.32 (0.88), [2.00-5.00]	3.81 (0.95), [2.00-5.00]	0.51 (0.10 - 0.92)*	0.57 (0.31 - 0.88)
14 [1]	Pay attention to your health needs?	4.64 (0.72), [2.00-5.00]	4.22 (0.99), [2.00-5.00]	0.41 (0.03 - 0.80)*	0.50 (0.29 - 0.82)
25 [1]	Find the energy to manage your health?	4.68 (0.67), [2.00-5.00]	4.19 (1.06), [1.00-5.00]	0.49 (0.10 - 0.88)*	0.57 (0.37 - 0.92)
27 [1]	Change your lifestyle to improve your health?	4.43 (0.87), [2.00-5.00]	4.00 (0.99), [2.00-5.00]	0.43 (0.02 - 0.85)*	0.47 (0.21 - 0.79)

The mean difference between the groups and associated effect size (Cohen's *d*) with 95% confidence intervals (CI) are included. The possible score range for each domain and item is 1-5.

* significant difference between No LBP and Chronic LBP groups.

## Discussion

To our knowledge this is the first study to utilise a quantitative tool to measure the broader elements of health literacy among community-dwelling individuals with CLBP and those with no history of LBP, and capture information which has predominantly been reported in qualitative investigations previously [21,25,26,36-38]. No differences were observed between the groups on seven of the eight domains of the instrument. Nonetheless, individuals with CLBP clearly experienced more difficulty in managing their health; that is *seeking and using *health information, within their current lifestyle (domain 1). These findings suggest that either overall personal management of health is the main health literacy area in which individuals with CLBP experience difficulty - a clinically important factor - or that the instrument was unable to detect other clinically important differences in health literacy between the groups.

Individuals are increasingly expected to take responsibility for their healthcare, particularly in the self-management of chronic conditions [10,39], including pain syndromes [40] and CLBP. Government policies and programmes are being developed and implemented to facilitate this process, particularly through models of care and models of health service delivery [41], including frameworks for LBP [42,43]. Although the reorientation of health services to support self-management is welcome, and based on evidence for the efficacy of self-management programmes for chronic conditions [44,45], our data highlight a situation where those health consumers who have a chronic condition, in this case CLBP, report greater difficulty in engaging in positive health behaviours compared to those individuals without LBP. This observation underlines the importance of self-management support and enablers for health behaviour change directed towards individuals with CLBP. It is also consistent with direct reports from patients with CLBP [36] and other musculoskeletal conditions [46], and mirrors the importance of self-management support for other chronic conditions [10,39].

Based on our data, individuals with CLBP had no greater difficulty in understanding health information (domain 2), engaging social support (domain 3), accessing healthcare (domain 4), accessing GP services (domain 5), communicating with health professionals (domain 6), being proactive (domain 7) or using health information (domain 8) than those individuals without LBP. Although our previous qualitative study highlighted that individuals with CLBP had difficulty in understanding anatomic and biomedical terms [21], in the current study they reported no greater difficulty than controls in understanding general health information. The absence of a group difference in this domain is likely due to the HeLMS items relating to general health information, whereas responses collected in the qualitative study related to specific biomedical terms used by health practitioners. Consistent with findings in the current study, participants in our earlier qualitative study also revealed no particular difficulty in engaging social support, communicating with health professionals or being proactive [21]. However, we did identify in the qualitative study that treatment costs were a barrier to utilising health information fully for individuals with CLBP [21]. Data from the current study demonstrate no difference in the ability to access health services between people with and without LBP, suggesting the capacity to access healthcare is comparable between the groups studied. Consistent with a lower score for domain 1, the score for each item within this domain was significantly lower among individuals with CLBP. The increased difficulties in making time for personal healthcare, paying attention to health needs, finding energy to manage health and optimising lifestyle habits are analogous to qualitative findings we reported previously among individuals with CLBP in the context of *utilising *health information [21], and other reports [36,38]. These also reflect barriers encountered by patients with other pain-related conditions such as rheumatoid arthritis [47], osteoarthritis [48] and spinal cord injury [49]. In our earlier qualitative study we found that a combination of personal and societal factors hindered utilisation of health information. Specifically, lifestyle commitments such as family responsibilities were cited as reasons for not engaging in positive health behaviours, such as exercise programmes. Participants also reported in the qualitative study that they could not incorporate behaviour changes into their current lifestyle due to lack of time and being unable to prioritise their health over other commitments [21].

The lower ability to engage in positive health behaviours by using health information among individuals with CLBP may represent an important factor in the aetiology or persistence of CLBP. For example, studies suggest that poor adherence to home exercise programmes, a key component of self-management for CLBP, mediates treatment efficacy for LBP [50,51] and contributes to sub-optimal recovery [36]. Suboptimal health behaviours may also relate to ineffective coping strategies for CLBP. A recent study identified that individuals with chronic pain who frequently used self-perceived ineffective coping strategies experienced more depression and pain-related anxiety compared to those who used effective coping strategies [52]. The authors suggested that a perceived lack of effective coping precludes individuals from engaging in more positive health behaviours, thereby perpetuating a cycle of chronic pain, ineffective coping and emotional distress. We cannot comment with certainty whether the difficulties experienced by participants with CLBP are causative for their pain experience or reflect sequelae of the increased personal burden associated with the experience of CLBP.

It is well established that patient beliefs and attitudes drive behaviour [21,53,54]. Therefore, efforts should be directed towards optimising LBP-related beliefs among individuals with CLBP in order to increase the likelihood of effective self-management behaviours. A range of factors influence self-management and health behaviour change including individual patient attributes, health professionals' beliefs and practice behaviours and health system functionality. Pain behaviours persist over time among individuals with CLBP, independent of pain severity and psychological factors, highlighting the difficulty in behaviour change and the need for a multifactorial approach [55]. Health behaviour change may only be observed once an individual decides on a readiness for change, identifies the importance of change and has the confidence to make change, although these phases may be facilitated with particular consultation skills [56,57]. Motivation and support from clinicians has been cited by patients with CLBP as critical to perseverance with self-management for CLBP [36] and other conditions [46]. Beliefs and attitudes held by clinicians are also critically important in a health behaviour change process [58]. The reluctance of the clinical community to adopt a patient-centred approach to CLBP management within a biopsychosocial model of care [59-63], consistent with best practice clinical guidelines, represents a key barrier to effective self-management and positive health behaviour change. At a system level, integration between health services, particularly in primary care, is also a critical element of health system reform needed to better support self-management in a flexible and patient-centred manner [39,64-66].

The health literacy characteristics highlighted in this study represent important barriers to adoption of positive health behaviours among individuals with CLBP. Clinicians should be cognizant of these health literacy components when expecting patients to engage in an extensive self-management programme for CLBP, particularly when programmes demand a large time or financial investment. For example, specific questions could be posed within a consultation to elucidate any barriers to seeking, understanding and utilising health information. In the context of pain syndromes, the Pain Stages of Change Questionnaire may be an appropriate tool to assess the extent to which individuals accept responsibility to engage in self management of their pain [67]. There is also evidence to suggest that self-management education for individuals with CLBP must be specific and individualised, rather than generic [68], since self-management and coping behaviours differ between subgroups of patients with chronic pain [52]. Successful implementation of health information into an individual's activities of daily living is likely to be contingent on personal attitudes towards their health in addition to family support, financial security and social support [10,11,38,69]. Care providers should therefore consider exploring these issues when expecting patients to undertake lifestyle changes as part of an overall management approach. The HeLMS may be an appropriate instrument to use in this context.

Despite the poorer back pain beliefs of the responding controls compared to the non-responding controls, significant differences in HeLMS scores were identified between the control and CLBP groups, which further supports the findings in this study. In the absence of a responder bias more differences between the groups may have been identified. We did not observe a difference in BBQ scores between back pain groups in this study. Whilst this is consistent with a recent study examining back pain beliefs in community-dwelling women [70], it is contrary to population-based surveys [71,72]. Recent data highlight that BBQ scores are influenced by level of pain, level of disability and impact of pain [21,70], and these factors may account for the absence of a group difference in this study.

Although we have reported statistically significant differences in HeLMS scores between the groups, the clinical significance remains uncertain. Future studies should establish the minimum clinically important difference (MCID) for the HeLMS domains and items. This would need to be explored across a range of normative and clinical populations. Further, this is the first time the questionnaire has been used in this setting and therefore a web of evidence is required before its utility can be judged fully. That is, further cohorts should be assessed with the HeLMS in order to assess consistency in any findings and aid in their interpretation. Although the response rate of this study was good (64-72%), the results should be interpreted within the context of some limitations. First, the study is limited by its cross-sectional design and relatively small sample resulting in power to only identify large effects. Second, our study population may not have been a truly representative sample of the Australian adult general population. The JSHS purposely recruited participants from the same middle-class geographical area, thereby minimising variability in socioeconomic status, an important correlate of health literacy [33]. Therefore, future studies should examine the potentially mediating influence of socioeconomic status on HeLMS scores. Third, all participants were middle-aged and utilised the internet at home, both factors associated with better functional health literacy scores compared with advanced age and lack of home internet access [33]. These factors were inclusion criteria for the JSHS. Fourth, all participants in the study were Australian residents and therefore health literacy characteristics should be interpreted within the context of the Australian primary health care setting.

Future studies should prospectively examine the effect of health literacy on the experience of back pain and determine whether the nature and history of the back pain experience, for example the duration, severity and particular functional impairments are related to particular health literacy skills and impairments. Difficulties experienced by individuals in the areas health attitudes and using health information should also be examined from a health provider perspective.

## Conclusions

There is no difference among individuals with CLBP and those without LBP among the majority of the broader elements of health literacy as measured by the HeLMS. However, adults with CLBP have greater difficulty in engaging in general positive health behaviours. This aspect of health literacy suggests that self-management support initiatives may benefit individuals with CLBP and this factor should be considered in clinical encounters.

## Competing interests

The author(s) declare that they have no competing interests.

## Authors' contributions

ABr was responsible for conception and design of the study, procurement of funding, data collection, data analysis and preparation of the manuscript. JJ and RB contributed to the design of the study, data collection and preparation of the manuscript. PO, ABu and LS contributed to the design of the study, procurement of funds and preparation of the manuscript. RO contributed to the preparation of the manuscript. All authors read and approved the final manuscript.

## Pre-publication history

The pre-publication history for this paper can be accessed here:

http://www.biomedcentral.com/1471-2474/12/161/prepub
